# Successful conversion surgery following chemotherapy with an immune checkpoint inhibitor in an older adult patient with stage IVB esophageal squamous cell carcinoma: a case report

**DOI:** 10.1186/s40792-023-01634-7

**Published:** 2023-03-30

**Authors:** Tetsuro Kawazoe, Shuhei Ito, Kippei Ohgaki, Yoshihiko Fujinaka, Hiroki Funakoshi, Akihiko Otake, Huanlin Wang, Kazutoyo Morita, Fumiyoshi Fushimi, Yoichi Ikeda

**Affiliations:** 1 Department of Gastrointestinal Surgery, Kyushu Central Hospital of the Mutual Aid Association of Public School Teachers, 3-23-1 Shiobaru, Minami-Ku, Fukuoka-City, Fukuoka 815-8588 Japan; 2 Department of Pathology, Kyushu Central Hospital of the Mutual Aid Association of Public School Teachers, Fukuoka, Japan

**Keywords:** Esophageal squamous cell carcinoma, Conversion surgery, Immunotherapy, Older patients

## Abstract

**Background:**

Chemotherapy and chemoradiotherapy are common treatments for esophageal squamous cell carcinoma with distant metastasis; however, the prognosis remains poor, and complete remission is difficult to achieve. Here, we report a case of an older adult patient with esophageal squamous cell carcinoma who underwent surgery following combined treatment of immunotherapy and chemotherapy and achieved pathological complete response.

**Case presentation:**

An 80-year-old woman presenting with difficulty swallowing was referred to our hospital. She was diagnosed with esophageal squamous cell carcinoma with distant metastasis of the lymph node at the dorsal side of the IVC and the left supraclavicular lymph node. She was treated with pembrolizumab, cisplatin, and 5-fluorouracil. After four pharmacotherapy courses, primary tumor and metastatic lymph node shrinkage was observed. The patient underwent thoracoscopic subtotal esophagectomy and regional lymph node dissection. The lymph node at the dorsal side of the IVC was not resected, and the left supraclavicular lymph node was removed. Histological examination revealed complete response with no residual tumor or lymph node metastasis. The patient had no recurrence 10 months postoperatively without adjuvant chemotherapy.

**Conclusions:**

Conversion surgery following preoperative therapy, including immunotherapy, may be an effective treatment strategy for improving survival in patients with esophageal squamous cell carcinoma even among older adult patients.

## Background

Esophageal cancer is a highly aggressive disease and is often diagnosed at a locally advanced or metastatic stage. It is the ninth most common cancer worldwide and the sixth leading cause of cancer-related death [1]. Squamous cell carcinoma accounts for the majority of the histological classification of esophageal cancer in Japan [2]. The primary treatment for esophageal squamous cell carcinoma (ESCC) without distant metastasis is surgery, which can include esophagectomy, radiation therapy, and chemotherapy. Conversion surgery following chemotherapy or chemoradiotherapy has been considered a promising strategy for improving the prognosis of patients with locally advanced ESCC [3].

Chemotherapy is the standard of care in cases of ESCC with distant metastasis, where the performance status is good and there is no obstruction. The combination of cisplatin and 5-fluorouracil (FP) has been considered the standard treatment for chemotherapy in cases of ESCC with distant metastasis [4]. To date, efficacy drugs in combination with FP therapy have been studied in several clinical trials. Particularly, the add-on effects of anti-epidermal growth factor receptor (EGFR) antibodies, such as cetuximab and panitumumab, have been studied; however, no superiority has been shown [5, 6]. In recent years, the efficacy of immune checkpoint inhibitors has been reported in several cancer types, and the efficacy of nivolumab, an anti-programmed death (PD)-1 antibody, has been demonstrated in the second-line treatment of esophageal cancer [7]. Furthermore, the KEYNOTE-590 and CheckMate 648 trials showed the superiority of the anti-PD-1 antibody and chemotherapy combination group over the chemotherapy alone [8, 9].

With the advancement of these drugs, cases of long-term survival and profound responses in ESCC with distant metastases have been observed. Conversely, there are limited reports on surgery following chemotherapy with immune checkpoint inhibitors. In this report, we describe a case of an older adult patient with stage IVB ESCC who underwent surgery following chemotherapy with an immune checkpoint inhibitor and pathologically confirmed complete response.

## Case presentation

**Fig. 1:**
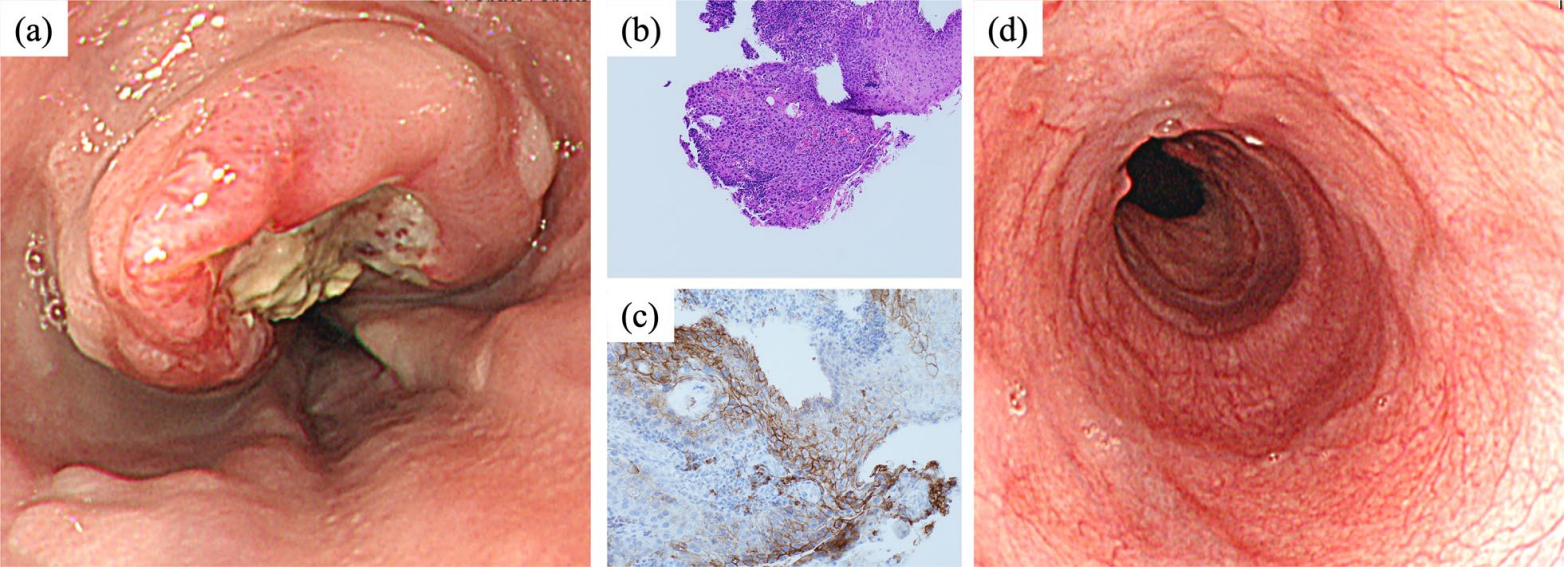
Upper endoscopy and pathological findings in the esophagus. Upper endoscopy reveals a half-circumferential ulcerative lesion in the thoracic lower esophagus at initial presentation (**a**). Biopsy specimen shows moderately differentiated squamous cell carcinoma (× 200) (**b**). The PD-L1 CPS is > 10% (× 200) (**c**). Following preoperative treatment, upper endoscopy reveals scarred mucosa in the area where the ulcerative lesion has been observed (**d**)

**Fig. 2:**
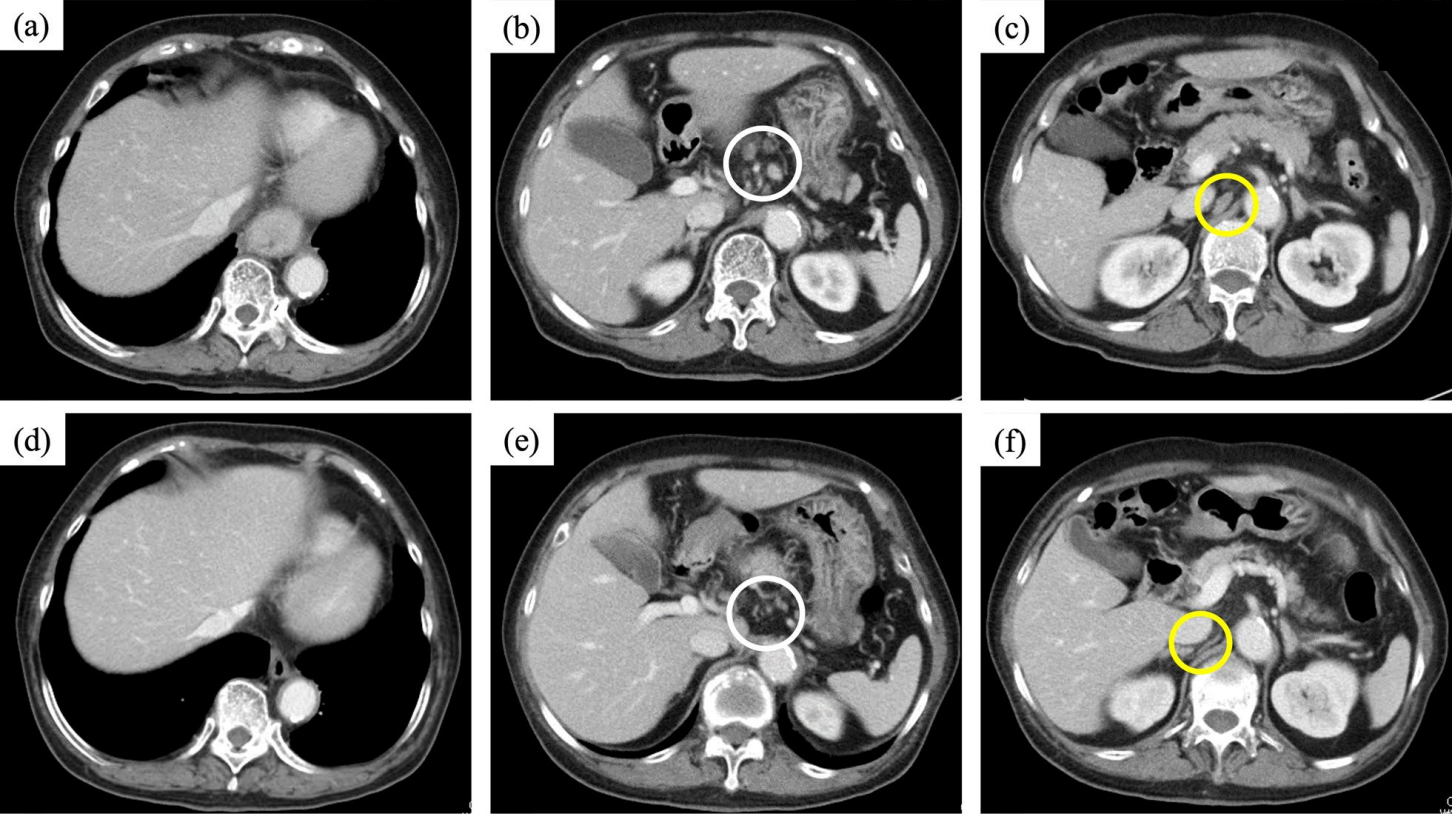
Enhanced computed tomography. Abdominal computed tomography reveals the thickening of the esophageal wall in the thoracic lower esophagus (**a**). The swelling of regional lymph nodes (**b**, white circle) and the lymph node on the dorsal surface of the inferior vena cava (**c**, yellow circle) is observed. Following preoperative treatment, the primary tumor (**d**), its regional lymph nodes (**e**, white circle), and the lymph node on the dorsal surface of the inferior vena cava (**f**, yellow circle) appear to shrink

**Fig. 3:**
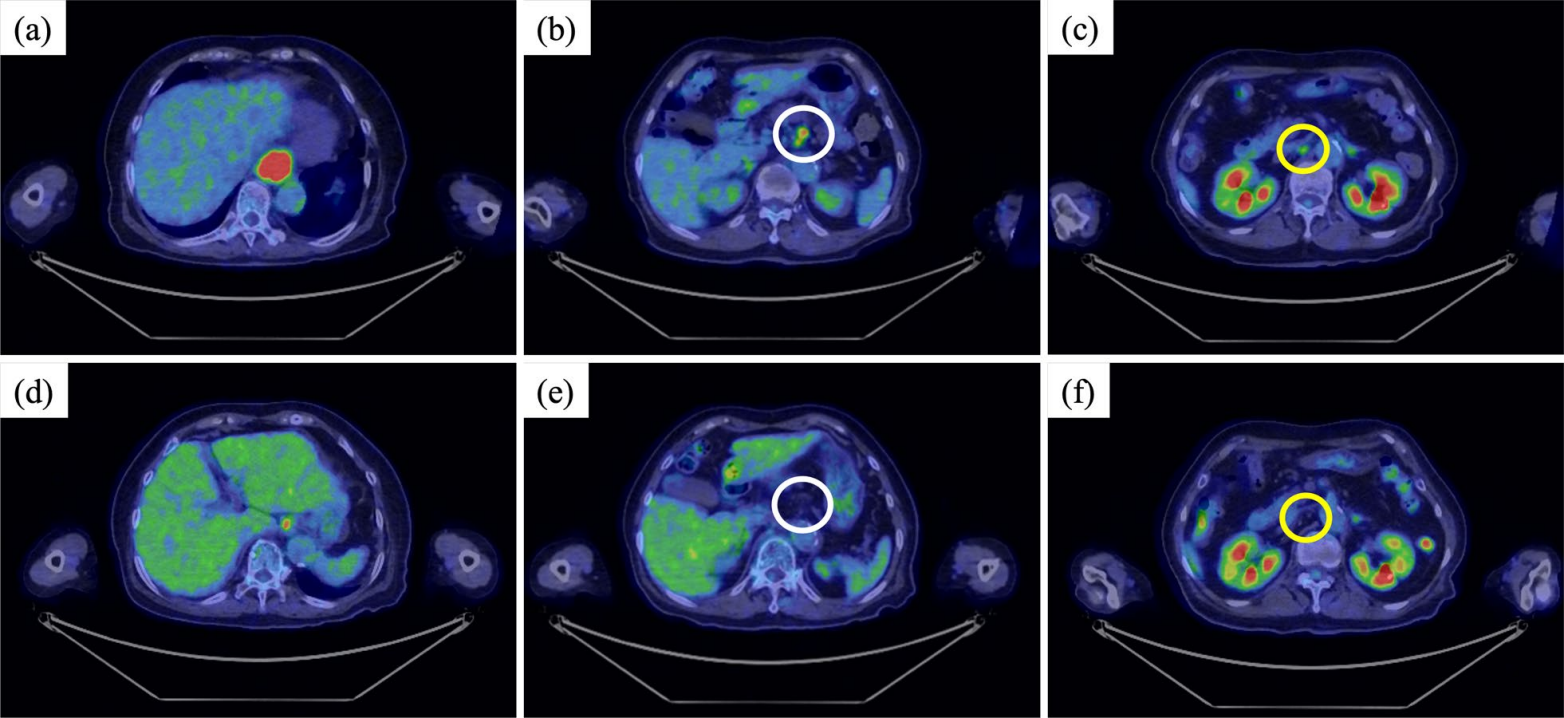
PET–CT. PET–CT reveals the abnormal FDG accumulation in the esophageal wall in the thoracic lower esophagus (**a**), regional lymph nodes (**b**, white circle), and the lymph node on the dorsal surface of the inferior vena cava (**c**, yellow circle). Following preoperative treatment, the abnormal FDG accumulation in the esophageal wall in the thoracic lower esophagus (**d**), regional lymph nodes (**e**, white circle) and the lymph node on the dorsal surface of the inferior vena cava (**f**, yellow circle) disappeared

An 80-year-old woman presenting with difficulty swallowing was referred to our hospital for further examination. Her Eastern Cooperative Oncology Group Performance Status was 0, and her serum creatinine level was 0.47 mg/dl indicating preserved renal function. Upper gastrointestinal endoscopy revealed a half-circumferential ulcerative lesion in the thoracic lower esophagus (Fig. 1a). Biopsy of the ulcerative lesion was performed, and a pathological diagnosis of moderately differentiated squamous cell carcinoma was made (Fig. 1b). The PD-ligand 1 (PD-L1) cell percentage score (CPS) of this specimen was > 10% (Fig. 1c). Computed tomography (CT) revealed the swelling of not only regional lymph nodes, including the perigastric lymph node, but also the left supraclavicular lymph node and the lymph node on the dorsal surface of the inferior vena cava (IVC) (Fig. 2a–c). Furthermore, the left supraclavicular lymph node and the lymph node on the dorsal surface of the IVC showed 18F-fluorodeoxyglucose (FDG) accumulation on positron emission tomography–CT (PET–CT) and were considered to be lymph node metastases (Fig. 3a–c). Consequently, the patient was clinically diagnosed with stage IVB ESCC (cT3N2M1, cStageIVB, Union for International Cancer Control [10] 8th edition TNM classification), and chemotherapy with pembrolizumab plus FP was planned. This regimen consisted of a 3-week course of 5-FU (800 mg/m^2^/day), intravenously administered on days 1–5, with cisplatin (80 mg/m^2^), intravenously administered on day 1, and pembrolizumab (200 mg/body), intravenously administered on day 1. In the first course, 5-FU and cisplatin were reduced to 80% dose considering her age. As there were no adverse events in the first course, 5-FU and cisplatin were administered at 100% dose in the second course. However, Grade 3 neutropenia was observed in the second course, so 5-FU and cisplatin were reduced to 80% dose in the third and fourth courses. After four courses of pembrolizumab plus FP therapy, upper gastrointestinal endoscopy revealed scarred mucosa in the area where the ulcerative lesion was observed (Fig. 1d). The biopsy specimen showed some atypical squamous cells with enlarged nuclei, suggesting a reactive change rather than a neoplastic lesion. CT revealed primary tumor and metastatic lymph node shrinkage (Fig. 2d–f), and PET–CT revealed no abnormal FDG accumulation in the lymph nodes where metastasis was originally suspected (Fig. 3d–f). Based on these findings, we determined the patient had downgraded to ycT2N0M0, ycStageII (UICC 8th edition) and that radical resection was feasible.

**Fig. 4:**
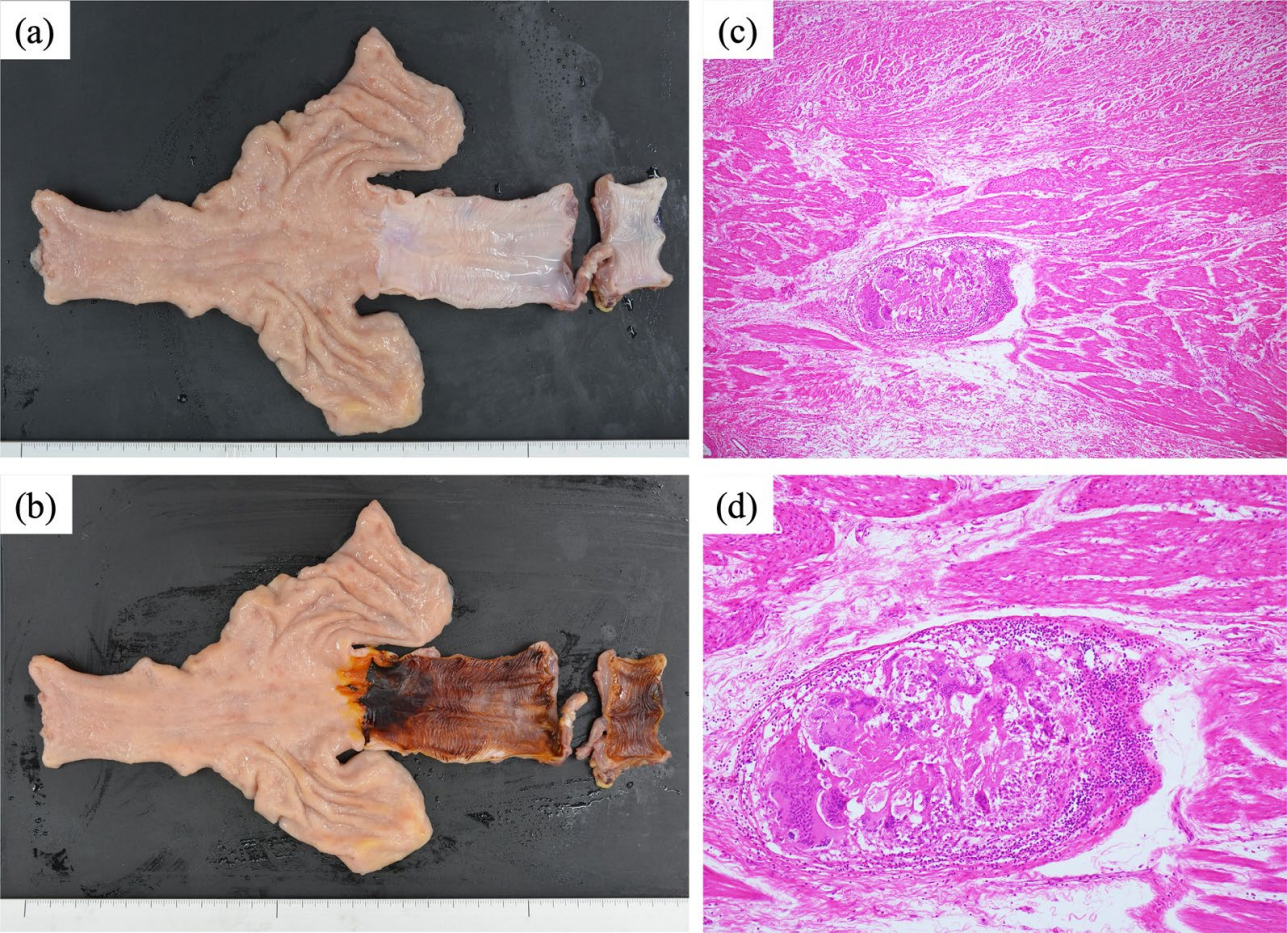
Histological findings. Macroscopically, an ulcerated scar measuring 18 × 5 mm at the site of the primary tumor is observed (**a**, **b**). Histological examination reveals no residual tumor (**c**, × 40) (**d**, × 100)

She subsequently underwent thoracoscopic subtotal esophagectomy reconstructed by a gastric tube retromediastinally with regional lymph node dissection. Considering the surgical invasiveness, the lymph node at the dorsal side of the IVC was not resected, and the left supraclavicular lymph node was removed. The resected specimen showed an ulcerated scar measuring 18 × 5 mm at the site of the primary tumor. Histological examination revealed no residual tumor, and a diagnosis of histological complete response (CR) was made (Fig. 4). No lymph node metastasis was observed in the regional lymph nodes. The postoperative course was uneventful, and the patient was discharged 16 days postoperatively. She received no adjuvant chemotherapy and remained alive without recurrence at 10 months postoperatively.

## Discussion

Although advances in multidisciplinary treatment have improved the outcome of esophageal cancer, the prognosis for stage IVB esophageal cancer remains poor [11]. In Japan, it is recommended that patients with stages II and III esophageal cancer should be treated with doublet or triplet preoperative chemotherapy followed by surgery [12, 13]. Chemoradiotherapy is recommended for locally advanced esophageal cancer (stage IVA) [14]. Recently, induction chemotherapy or chemoradiotherapy followed by surgery has become a widely used strategy for the treatment of locally advanced esophageal cancer [15, 16]. Immunotherapy has been introduced in esophageal cancer, and preoperative treatment with immunotherapy has been shown to be safe and feasible for locally advanced esophageal cancer [17, 18]. However, treatment strategies for stage IVB esophageal cancer with distant metastasis are limited to chemotherapy and chemoradiotherapy, and the usefulness of conversion surgery has not been clarified.

In this report, we described a patient with stage IVB ESCC. She was diagnosed with stage IVB ESCC based on the enlarged lymph nodes at the dorsal side of the IVC and above the left clavicle at initial presentation. The patient was 80 years old; however, her performance status was preserved. She was started on palliative chemotherapy for unresectable esophageal cancer. Pembrolizumab plus FP was selected as the first-line regimen based on her high PD-L1 CPS (> 10%). The patient completed four courses of the treatment regimen without any grade 3 or higher adverse events of Common Terminology Criteria for Adverse Events. After four courses of immunotherapy and chemotherapy, since imaging studies revealed metastatic lymph node shrinkage and that radical resection was feasible, we planned to perform a conversion surgery.

Using the PubMed database using the terms “esophageal cancer” and “conversion surgery”, no reports on conversion surgery following combined treatment of immunotherapy and chemotherapy for initially unresectable esophageal cancer due to distant metastasis were noted; however, some reports on conversion surgery for initially unresectable esophageal cancer due to adjacent organ invasion were noted [17, 19]. To our knowledge, this is the first report on conversion surgery following combined treatment of immunotherapy and chemotherapy in patients with initially unresectable esophageal cancer due to distant metastasis.

The efficacy of immune checkpoint inhibitors as preoperative therapy has been demonstrated in several cancer types, including melanoma, non-small cell lung cancer, and even mismatch repair-proficient and mismatch repair-deficient colon cancers [20–22]. In the field of upper gastrointestinal cancer, Kumamoto et al. reported a case of esophagogastric junction cancer with para-aortic lymph node metastasis that was treated with nivolumab followed by surgery with confirmed CR [23], and Matsumoto et al. reported a case of gastric cancer with multiple liver metastases that was treated with nivolumab followed by surgery with confirmed CR [24], though nivolumab was administered as the third-line therapy in the above cases. In our case, the PD-L1 CPS was high (> 10%), which predicted the efficacy of immunotherapy. However, because the pembrolizumab and platinum agent combination has been suggested to be effective even in the microsatellite-stable population in ESCC [25], further investigation is needed regarding the indication of immunotherapy as a preoperative therapy.

In this case, we performed conversion surgery after four cycles of pharmacotherapy. For locally advanced unresectable ESCC, conversion surgery after 2–3 cycles of induction chemotherapy is becoming considered. Yokota et al. reported the efficacy of 3 cycles of induction chemotherapy using docetaxel, cisplatin, and 5-FU followed by conversion surgery [26] and Huan et al. reported the feasible result of conversion surgery following at least 2 cycles of immunochemotherapy [27] for initially unresectable locally advanced ESCC. The optimal duration period of chemotherapy prior to conversion surgery for ESCC with distant metastasis is unclear. Concerning about gastric cancer, Yoshida et al. estimated the best timing for the removal of the tumor is 4–6 cycles of chemotherapy in gastric cancer with distant metastasis [28], on the other hand, there have been reported cases of conversion surgery performed after 10 or more cycles of chemotherapy [29, 30]. It is difficult to determine whether the tumor can be controlled through surgical resection or whether they have already developed into a systemic disease, and further investigation is needed to determine the optimal treatment period before conversion surgery.

The number of older adult patients with esophageal cancer is increasing. Older adult patients frequently have decreased tolerability to chemotherapy and surgery [31]. Although the JCOG9907 trial demonstrated the efficacy of preoperative chemotherapy in patients 75 years old or younger [12], Suzuki et al. reported that preoperative chemotherapy was also effective in patients 76 years old or older [32]. Although thoracoscopic esophagectomy was reported to be safely performed in older adult patients, the prognosis was poor compared with that in non-older adult patients [33]. Although preoperative chemotherapy has been reported to be ineffective in patients over the age of 75 [34], and chemotherapy with docetaxel is poorly tolerated among older adults [35], the KEYNOTE-590 trial did not exclude patients simply because they were older, suggesting that immunotherapy may be a beneficial treatment for older adults. Efficacy of immune checkpoint inhibitors has been shown among patients aged 80 years or older in an international cohort study [36].

No signs of recurrence were observed 9 months postoperatively. Although the efficacy of conversion surgery following chemotherapy for ESCC with distant metastasis has not been established, the metastatic lymph node shrinkage and the pathological CR in the resected specimen following combined treatment of immunotherapy and chemotherapy were confirmed in the present case, suggesting that it may be considered in older adult patients if their general condition is well maintained, but careful consideration is required in determining its indications.

## Conclusions

We have demonstrated a case of a patient with stage IVB ESCC with distant metastasis who achieved pathological CR following preoperative treatment with immunotherapy and chemotherapy. We suggest that conversion surgery following preoperative therapy, including immunotherapy, has clinical utility for the treatment of stage IVB ESCC even among older adult patients.

## Data Availability

Data sharing is not applicable to this article, as no datasets were generated or analyzed during the current study.

## Abbreviations

ESCC: Esophageal squamous cell carcinoma
FP: Cisplatin and 5-fluorouracil
EGFR: Epidermal growth factor receptor
PD-1: Programmed death-1
PD-L1: Programmed death-ligand 1
CPS: Cell percentage score
CT: Computed tomography
IVC: Inferior vena cava
FDG: 18F-Fluorodeoxyglucose
PET–CT: Positron emission tomography–CT
UICC: Union for International Cancer Control
CR: Complete response
